# How to Stop Hiccough

**Published:** 1909-10

**Authors:** 


### Abstracts and Selections.

### How to Stop Hiccough.

This is often a very serious matter, and, as is well known, sometimes impossible. Hiccough is usually of no consequence, but during the course of acute diseases it becomes frequently a very dangerous and difficult complication. In the *Maryland Medical Journal* Dr. Kolopinski says he was able to stop the hiccough in such a case by taking a large spoon handle and pressing the tongue down and back with a steady force. He continued the pressure on the tongue, with the hope of further knowing the action of the palate muscles, when, to his surprise, the hiccoughs ceased. After the doctor's departure the hiccoughs returned, and the patient applied the spoon handle himself to the back of the tongue and with both hands pushed down firmly. The hiccough again ceased. The hiccough appeared several times later, and was always promptly stopped by the application of the spoon handle.

This is a very valuable suggestion, as it can be used by anybody, and might be the means of saving many lives.—*Southern California Practitioner.*

